# Defining proficiency in THD-Anolift: a CUSUM analysis of the learning curve in 51 consecutive cases

**DOI:** 10.1007/s00384-026-05151-5

**Published:** 2026-05-20

**Authors:** Andrea Cesare Galli, Gianlorenzo Dionigi, Sara Lauricella, Sergio Agradi, Richard Sassun, Angelo Emanuele Leone, Roberto Cirocchi, Francesco Brucchi

**Affiliations:** 1 Division of General Surgery, Istituto Auxologico Italiano, IRCCS (Istituto Di Ricovero E Cura a Carattere Scientifico), Milan, Italy; 2 Department of Pathophysiology and Transplantation, University of Milan, Milan, Italy; 3 Policlinico San Pietro, Unit of Colon Proctology and Pelviperineology, Ponte San Pietro, Bergamo Italy; 4 General Surgery Unit, Pio XI Hospital, Desio, Monza e Brianza Italy; 5 Department of Medicine and Surgery, University of Perugia, 06129 Perugia, Italy

**Keywords:** Hemorrhoidal disease, Transanal hemorrhoidal dearterialization, THD, Anolift, Learning curve, CUSUM, Mucopexy, Surgical training

## Abstract

**Background:**

Transanal hemorrhoidal dearterialization (THD) with Anolift mucopexy is a validated non-excisional procedure for hemorrhoidal disease. Although surgeon experience is acknowledged as a determinant of THD outcomes, no formal learning curve analysis exists. This study aimed to characterize the learning curve of a single surgeon adopting THD-Anolift.

**Methods:**

Retrospective analysis of 60 consecutive THD-Anolift cases (May 2023–February 2026). Nine patients with incomplete outcome data were excluded, leaving 51 for analysis. Median follow-up was 12 months (range 3–33). Cumulative sum (CUSUM) charts were constructed for a composite failure endpoint (recurrence and/or any complication) and for operative time. The proficiency point was identified at the CUSUM inflection. Sensitivity analyses included CUSUM on recurrence alone, CUSUM restricted to recurrence and Clavien-Dindo ≥ II complications, best-case/worst-case imputation for excluded patients, and risk-adjusted CUSUM controlling for hemorrhoid grade and previous procedures.

**Results:**

Median age was 53 years; 72.5% were male; 80.4% had grade III hemorrhoids. The overall composite failure rate was 37.3% (19/51) and the recurrence rate 15.7% (8/51). Mean operative time was 23.5 ± 7.4 min. The composite outcome CUSUM identified a proficiency point at case 23: the failure rate decreased from 52.2% in Phase 1 (cases 1–23) to 25.0% in Phase 2 (cases 24–51; *p* = 0.080, not statistically significant at the conventional threshold). Operative time decreased from 27.1 ± 7.4 to 20.4 ± 6.0 min (*p* < 0.001). On sensitivity analysis, both the recurrence-only CUSUM and the risk-adjusted CUSUM confirmed an identical proficiency point at case 23, and worst-case/best-case imputation for excluded patients moved the inflection only to cases 25 and 22 respectively, indicating that the finding was robust to endpoint definition and case-mix variation, as well as to plausible patterns of missing data.

**Conclusions:**

CUSUM analysis identified an inflection at approximately 23 cases, with a statistically significant reduction in operative time and a clinically relevant but underpowered reduction in composite failure that did not reach the conventional threshold for statistical significance. Rather than a fixed competency threshold, these findings provide an initial benchmark to help structure supervised adoption—suggesting that the 10 mentored cases conventionally proposed in industry-sponsored teaching may be insufficient— and to inform future multicenter validation.

## Introduction

Hemorrhoidal disease (HD) is one of the most common proctologic conditions, affecting approximately one-third of the general population and frequently impairing quality of life [1, 2]. Over the past two decades, non-excisional surgical approaches have gained popularity for their reduced postoperative pain, faster recovery, and preservation of anorectal anatomy compared with conventional hemorrhoidectomy [3, 4].

Transanal Hemorrhoidal Dearterialization (THD), originally described by Morinaga et al. [5], combines Doppler-guided ligation of terminal branches of the superior rectal artery with mucopexy, addressing both vascular hyperflow and mucosal prolapse [6]. The Anolift modification employs a unidirectional barbed suture to distribute tension evenly along the plication line, simplifying the management of large prolapsing hemorrhoids and potentially reducing postoperative tenesmus [7, 8]. Clinical guidelines from the ASCRS and the Italian SICCR recognize THD as a validated option for grades II–III and selected grade IV hemorrhoids [9–11].

While comparative data consistently demonstrate favorable perioperative outcomes for THD [3, 12–14], recurrence rates remain higher than after excisional procedures. Several authors have suggested that surgeon experience may be a key determinant of THD outcomes [8, 14–16]. The HubBLe trial noted that a learning curve longer than the manufacturer-recommended 10 mentored cases may exist, but acknowledged the absence of data to define procedural competence [14]. A meta-analysis by Emile et al. [13] reported 6% technical failure after THD, further underscoring the role of training. Despite these observations, no formal learning curve analysis for THD or THD-Anolift has been published to date.

The aim of this study was to characterize the learning curve of THD-Anolift performed by a single surgeon using cumulative sum (CUSUM) analysis, evaluating trends in operative time and a composite clinical outcome across consecutive cases.

## Methods

This was a retrospective cohort study of all consecutive patients who underwent THD with Anolift mucopexy performed by a single surgeon (AG) at IRCCS Istituto Auxologico Italiano, Milan, Italy between May 2023 and February 2026. The surgeon had no prior independent experience with THD, making this a true inception-of-technique analysis. The study followed STROBE guidelines for observational research [17] and was approved by the institutional ethics committee. All patients provided informed consent for surgery and anonymized data use.

Inclusion criteria were: age ≥ 18 years, symptomatic HD grade II–IV (after failure of conservative management), and availability of follow-up data on recurrence and complications. Exclusion criteria were: acute thrombosed or fibrotic hemorrhoids, anorectal sepsis, inflammatory bowel disease, and incomplete data on the primary outcome variables. Patients with not-reported (NR) values for recurrence, complications, or follow-up were excluded from the analysis.

All procedures were performed as day-case surgery under spinal anesthesia, in lithotomy position. The technique has been described in detail by Giordano and Schembari [8]. Briefly, the dearterialization phase used a THD Slide™ proctoscope (THD Lab, Correggio, Italy) with Doppler-guided identification and Z-suture ligation of all terminal branches of the superior rectal artery. The mucopexy phase used a synthetic absorbable monofilament (Polydioxanone) 2/0 barbed suture (Filbloc, Assut Europe) on a 4/8 30 mm needle, applied as a continuous running suture from the proximal rectum distally, stopping ≥ 5 mm from the dentate line. The barbed suture allows self-locking plication without a terminal knot, distributing tension evenly and avoiding the pocket effect associated with conventional mucopexy [8].

Data were extracted from a prospectively maintained database. Variables included demographics (age, sex, ASA score), Goligher grade, history of previous anorectal procedures, operative time (insertion to removal of proctoscope), number of dearterialization points, mucopexy performed and number of plications, length of hospital stay, early postoperative complications, late complications at follow-up, recurrence (bleeding, prolapse, or both), and reoperation.

The primary outcome was a composite binary failure endpoint: occurrence of recurrence and/or any complication (postoperative or at follow-up). For descriptive purposes, complications were also categorised post hoc according to the Clavien-Dindo classification, distinguishing self-limiting events not requiring specific therapy (grade I, e.g., transient tenesmus, mild anal pain) from events requiring pharmacological intervention or reoperation (grade ≥ II), and a recurrence-and-major-complication endpoint was examined as a complementary analysis. The secondary outcome was operative time as a continuous measure of technical efficiency.

Pre-specified sensitivity analyses included: (a) CUSUM on recurrence alone, to assess whether the proficiency point was driven by minor complications; and (b) risk-adjusted CUSUM (RA-CUSUM), in which the expected probability of failure for each case was estimated by logistic regression including hemorrhoid grade IV (binary) and previous procedures (binary) as covariates, to account for potential case-mix variation between phases.

Patients were ordered chronologically by date of surgery. For the composite outcome, the CUSUM at case i was calculated as: CUSUM(i) = CUSUM(i − 1) + [X(i) – p0], where X(i) is the outcome (1 = failure, 0 = success) and p0 is the overall observed failure rate. The peak of the resulting curve identifies the proficiency point, i.e. the case after which performance was consistently better than the cohort average [18, 19]. For operative time: CUSUM(i) = CUSUM(i − 1) + [T̅ – T(i)], where T(i) is individual operative time and T̅ the overall mean; the trough marks the inflection toward faster procedures.

For the RA-CUSUM: CUSUM(i) = CUSUM(i − 1) + [X(i) – p(i)], where p(i) is the patient-specific predicted failure probability from the logistic model. This adjusts the learning curve for case complexity [18].

Based on the composite outcome CUSUM inflection, the cohort was divided into Phase 1 (pre-proficiency) and Phase 2 (post-proficiency). Continuous variables were compared using the Mann–Whitney U test; categorical variables using Fisher exact test. Tertile analysis was performed as a complementary approach (Kruskal–Wallis, chi-square for trend). Segmented linear regression modeled operative time trends before and after the proficiency point. Follow-up duration was calculated from the date of surgery to the most recent outpatient or telephone contact, and reported as median with range for the overall cohort and by phase. To address the potential impact of excluded patients on the temporal continuity of the CUSUM, we (i) tabulated the chronological position of each excluded case along the surgical sequence, (ii) compared baseline characteristics between included (*n* = 51) and excluded (*n* = 9) patients, and (iii) performed best-case/worst-case imputation, in which excluded patients were assumed in turn to have had no failure or to have failed, and the CUSUM was re-computed under each scenario to assess the stability of the proficiency point. Statistical significance was set at *p* < 0.05 (two-tailed). Analyses used Python 3.11 (SciPy 1.11, scikit-learn 1.3).

## Results

**Fig. 1:**
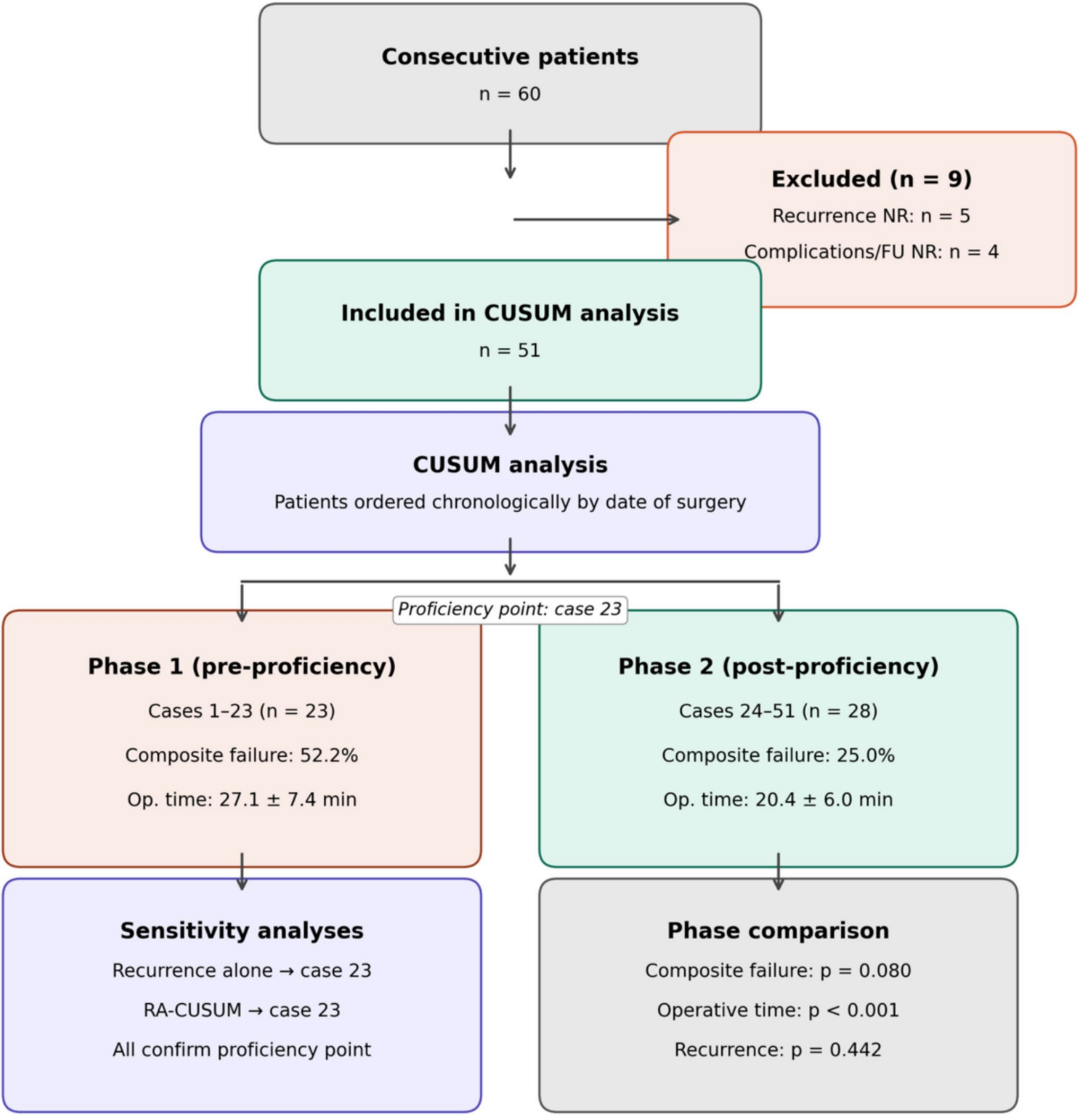
Study flow diagram. Of 60 consecutive THD-Anolift patients, 9 were excluded for incomplete outcome data. The remaining 51 were ordered chronologically and analyzed by CUSUM

Of 60 consecutive patients, 9 (15%) were excluded for incomplete data on recurrence (*n* = 5), complications (*n* = 4), or follow-up date (*n* = 9, overlapping). By chronological position in the operative sequence, the 9 excluded cases were not concentrated in the early phase: 2 occurred among the first 20 procedures, 3 among procedures 21–40, and 4 among the last 20 procedures, with the median rank of excluded cases being 35 (range 4–58). Baseline characteristics of excluded patients (median age 51 years, 66.7% male, 88.9% grade III, 11.1% with previous anorectal procedures) did not differ materially from those of the analyzed cohort, and no grade IV patient was lost to follow-up. Fifty-one patients were analyzed (Fig. 1). Median age was 53 years (range 21–85), 37 (72.5%) were male, and the ASA distribution was I in 47.1%, II in 47.1%, and III in 5.9%. Hemorrhoid grade was III in 41 patients (80.4%), IV in 5 (9.8%), II in 3 (5.9%), and unrecorded in 2 (3.9%). Nine patients (17.6%) had prior anorectal procedures. Mucopexy was performed in 44 (86.3%), with a mean of 1.9 plications. Nearly all patients (96.1%) were discharged on postoperative day 1. The median follow-up was 12 months (range 3–33) overall, 18 months (range 6–33) in Phase 1 and 8 months (range 3–18) in Phase 2, reflecting the longer observation window available for earlier procedures. Baseline characteristics were comparable between phases for age, sex, and ASA score. However, all 5 grade IV patients were operated during Phase 1, and a higher proportion had prior anorectal procedures (30.4% vs. 7.1%; p = 0.060), resulting in a significantly different grade distribution (*p* = 0.010; Table 1). This imbalance is addressed in the sensitivity analyses.

**Table 1:** Patient characteristics by learning curve phase

	Overall (*n* = 51)	Phase 1 (*n* = 23)	Phase 2 (*n* = 28)	p
Age, years, median (range)	53 (21–85)	53 (21–81)	50 (26–85)	0.977^1^
Male sex, n (%)	37 (72.5)	16 (69.6)	21 (75.0)	0.757^2^
ASA score, n				0.915^3^
I	24 (47.1)	11 (47.8)	13 (46.4)	
II	24 (47.1)	11 (47.8)	13 (46.4)	
III	3 (5.9)	1 (4.3)	2 (7.1)	
Goligher grade, n				0.010^2^†
II	3 (5.9)	0 (0)	3 (10.7)	
III	41 (80.4)	16 (69.6)	25 (89.3)	
IV	5 (9.8)	5 (21.7)	0 (0)	
Not recorded	2 (3.9)	2 (8.7)	0 (0)	
Previous anorectal procedures, n (%)	9 (17.6)	7 (30.4)	2 (7.1)	0.060^2^
Mucopexy performed, n (%)	44 (86.3)	21 (91.3)	23 (82.1)	0.436^2^
No. of plications, mean ± SD	1.9 ± 0.7	2.0 ± 0.8	1.7 ± 0.7	0.289^1^
Hospital stay = 1 day, n (%)	49 (96.1)	22 (95.7)	27 (96.4)	1.000^2^

Phase 1 = cases 1–23 (pre-proficiency); Phase 2 = cases 24–51 (post-proficiency). ^1^Mann-Whitney U. ^2^Fisher exact. ^3^Chi-square. †All grade IV patients were operated in Phase 1; see Discussion

Mean operative time was 23.5 ± 7.4 min (range 10–40). Eight patients (15.7%) developed recurrence: 4 with bleeding, 3 with prolapse, and 1 with both. Fifteen patients (29.4%) had at least one complication: tenesmus (*n* = 7, 13.7%) was the most frequent, followed by anal pain (*n* = 5, 9.8%) and bleeding (*n* = 4, 7.8%), with 2 patients reporting multiple symptoms. According to the Clavien-Dindo classification, the majority of complications were grade I (transient tenesmus and mild anal pain managed with oral analgesia, *n* = 11/15), while 4 patients (7.8%) experienced grade II events (postoperative bleeding requiring outpatient haemostatic therapy without transfusion or reintervention) and 1 patient (2.0%) had a grade IIIb event requiring reoperation for recurrent prolapse. When the composite endpoint was restricted to recurrence and Clavien-Dindo ≥ II events (i.e., excluding self-limiting grade I complications), the overall failure rate was 23.5% (12/51), decreasing from 34.8% in Phase 1 to 14.3% in Phase 2 (Fisher exact *p* = 0.105). The composite failure endpoint occurred in 19 patients (37.3%). One patient (2.0%) required reoperation.

**Fig. 2:**
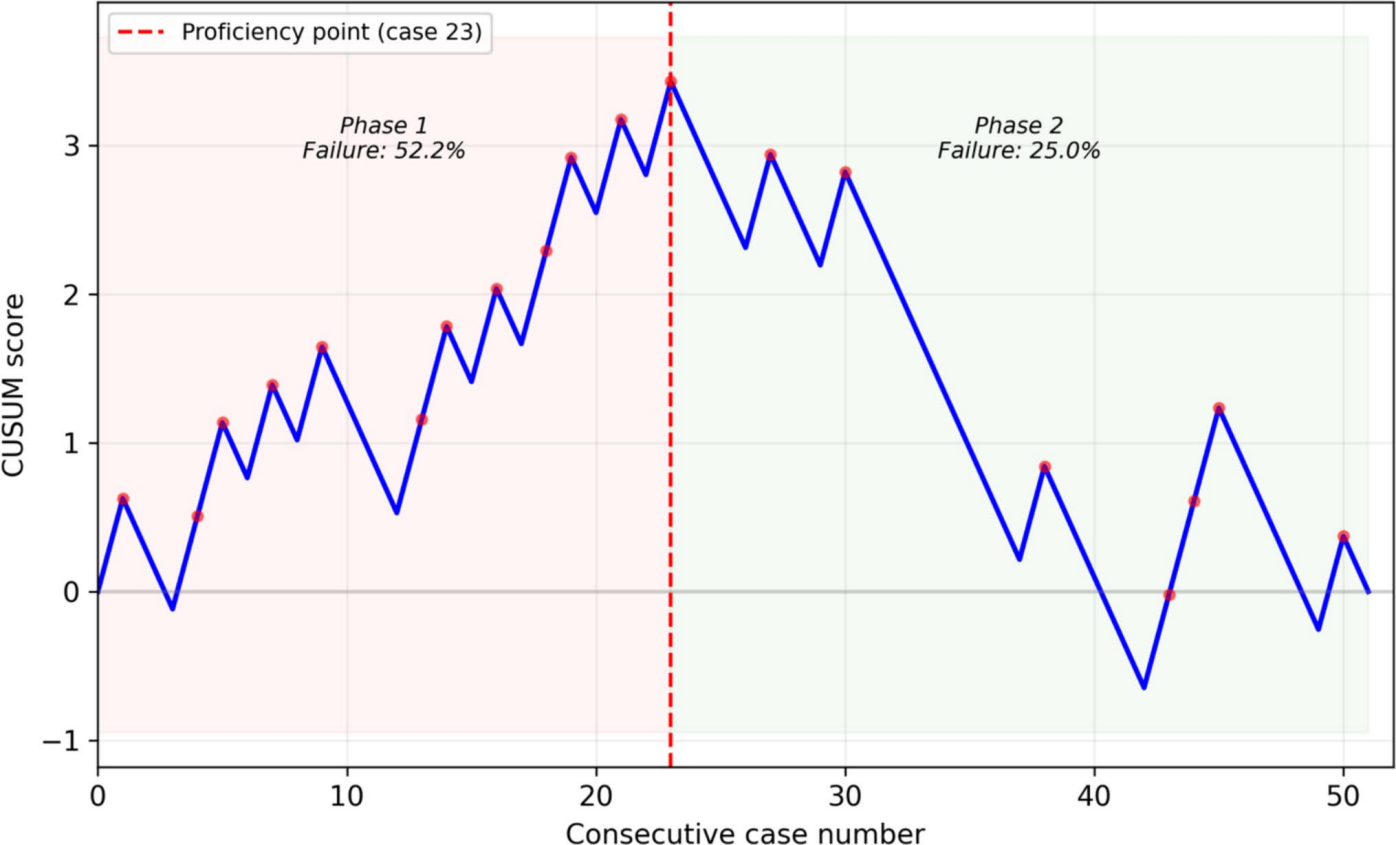
CUSUM chart for the composite failure endpoint. The curve peaks at case 23 (dashed red line), defining the proficiency point. Red dots mark individual failure events. Shading: Phase 1 (light red, pre-proficiency) and Phase 2 (light green, post-proficiency). p0 = 37.3% (overall observed failure rate)

The CUSUM chart (Fig. 2) rose steeply during the initial cases and peaked at case 23, defining the proficiency point. In Phase 1 (cases 1–23, *n* = 23), the composite failure rate was 52.2% (12/23); in Phase 2 (cases 24–51, *n* = 28), it was 25.0% (7/28; OR 3.27; Fisher exact *p* = 0.080). The complication rate decreased from 43.5% to 17.9% (*p* = 0.066) and the recurrence rate from 21.7% to 10.7% (*p* = 0.442).

**Fig. 3:**
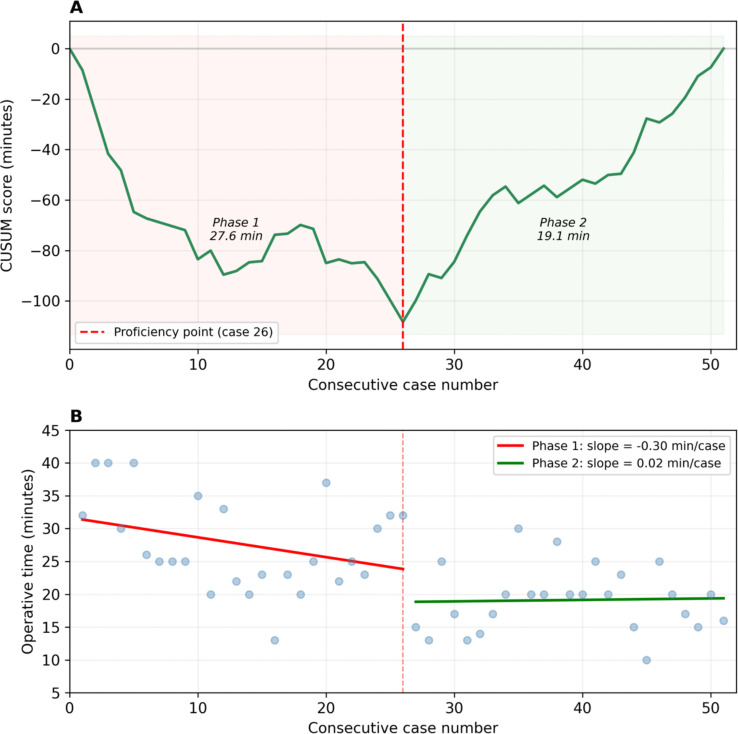
(**A**) CUSUM chart for operative time, with trough at case 26 confirming a concordant proficiency point. (**B**) Individual operative times with segmented regression: Phase 1 slope =  − 0.58 min/case (*p* = 0.009); Phase 2 slope =  − 0.23 min/case (*p* = 0.098, plateau)

The operative time CUSUM identified a concordant proficiency point at case 26 (Fig. 3A). At the primary cutoff of case 23, mean operative time was 27.1 ± 7.4 min in Phase 1 versus 20.4 ± 6.0 min in Phase 2, a reduction of 6.7 min (24.7%; Mann–Whitney *p* < 0.001). Segmented regression showed a significant learning slope of − 0.58 min/case in Phase 1 (*p* = 0.009) that flattened in Phase 2 (− 0.23 min/case, p = 0.098), consistent with stabilization (Fig. 3B). Overall linear regression confirmed a significant downward trend (− 0.29 min/case, *p* < 0.001).

**Fig. 4:**
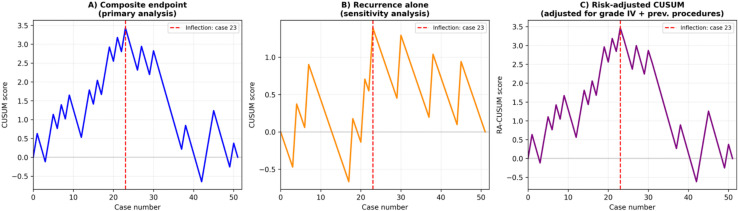
Sensitivity analyses. (**A**) Standard CUSUM on composite endpoint (primary, inflection case 23). (**B**) CUSUM on recurrence alone (inflection case 23). (**C**) Risk-adjusted CUSUM, controlling for hemorrhoid grade and previous procedures (inflection case 23). All three analyses identify an identical proficiency point, confirming robustness

Three sensitivity analyses were performed (Fig. 4). First, the CUSUM on recurrence alone (excluding all complications) identified an identical proficiency point at case 23. The recurrence rate decreased from 21.7% in Phase 1 to 10.7% in Phase 2. Second, the risk-adjusted CUSUM, which accounted for the uneven distribution of grade IV hemorrhoids and previous procedures between phases, also yielded an inflection at case 23. The logistic model showed that these covariates had negligible predictive power (expected failure probabilities ranged from 34.2% to 40.1%), confirming that baseline case complexity did not fully explain the observed learning effect. Given the limited sample size (51 patients, 19 events), this adjustment should be interpreted as exploratory. Third, tertile analysis showed a significant stepwise decrease in operative time across experience (27.8, 22.4, 20.2 min; Kruskal–Wallis *p* = 0.011) and a decreasing trend in composite failure (47.1%, 35.3%, 29.4%; chi-square *p* = 0.556; Table 2).

**Table 2:** Outcomes by learning curve phase and tertile

Phase comparison	Phase 1 (*n* = 23)	Phase 2 (*n* = 28)	p	Overall
Composite failure, n (%)	12 (52.2)	7 (25.0)	0.080	19 (37.3)
Recurrence	5 (21.7)	3 (10.7)	0.442	8 (15.7)
Any complication	10 (43.5)	5 (17.9)	0.066	15 (29.4)
Operative time, min (± SD)	27.1 ± 7.4	20.4 ± 6.0	< 0.001	23.5 ± 7.4
Reoperation, n (%)	1 (4.3)	0 (0)	–	1 (2.0)
**Tertile comparison**	**T1 (n = 17)**	**T2 (n = 17)**	**T3 (n = 17)**	**p-trend**
Composite failure, n (%)	8 (47.1)	6 (35.3)	5 (29.4)	0.556
Operative time, min	27.8	22.4	20.2	0.011
Recurrence, n (%)	2 (11.8)	4 (23.5)	2 (11.8)	NS

Phase: Fisher exact (categorical), Mann–Whitney U (continuous). Tertile: chi-square for trend, Kruskal–Wallis. NS = not significant

A fourth, post hoc sensitivity analysis addressed the potential influence of the 9 patients excluded for incomplete data on the temporal continuity of the CUSUM. Imputation of all excluded patients as failures (worst-case scenario) shifted the CUSUM peak only marginally (from case 23 to case 25), while imputation as non-failures (best-case scenario) shifted it from case 23 to case 22. Across both extremes the inflection therefore remained within a narrow window of cases 22–25, supporting the robustness of the estimated proficiency point to plausible patterns of missingness. Restricting the composite endpoint to recurrence and Clavien-Dindo ≥ II events also produced an inflection at case 23, with Phase 1 vs. Phase 2 rates of 34.8% vs. 14.3% (*p* = 0.105) Table 3.

**Table 3:** Sensitivity analyses: proficiency point consistency

Analysis	Proficiency point	Phase 1 rate	Phase 2 rate	p
Composite outcome (primary)	Case 23	52.2%	25.0%	0.080
Recurrence alone	Case 23	21.7%	10.7%	0.442
Risk-adjusted CUSUM*	Case 23	52.2%	25.0%	0.080
Operative time	Case 26	27.1 min	20.4 min	< 0.001

^*^Exploratory adjustment for grade IV hemorrhoids and previous procedures via logistic regression (51 patients, 19 events)

## Discussion

This study provides the first formal learning curve analysis for THD-Anolift. CUSUM analysis identified a proficiency point at approximately 23 cases, with a concurrent statistically significant improvement in operative efficiency and a favorable but underpowered trend in composite clinical outcomes that did not reach conventional statistical significance (*p* = 0.080). Importantly, sensitivity analyses confirmed that this inflection was robust to endpoint definition, persisting when only recurrence was considered, when self-limiting Clavien-Dindo grade I events were excluded, and after risk-adjustment for case complexity, as well as under best-case/worst-case imputation for excluded patients.

The concordance between the outcome-based (case 23) and time-based (case 26) proficiency points suggests that technical efficiency and clinical decision-making mature in parallel for this procedure. This pattern differs from procedures where operative time improves well before clinical outcomes [18, 19], and may reflect the tight coupling between suture technique and clinical results in THD-Anolift.

The inflection observed at approximately 23 cases should not be interpreted as a universal threshold for proficiency, but rather as an empirically derived estimate from a single-surgeon inception series. Learning curves are likely to vary according to prior proctologic experience, case selection, mentorship, and institutional setting. Nevertheless, these data carry an explicit training implication. The 10 mentored procedures commonly cited in industry-sponsored teaching materials and reflected in the HubBLe trial protocol [14] would correspond, in the present series, to a phase in which both operative time and the composite failure rate were still well above their post-proficiency values: at case 10 the cumulative failure rate was 60% and the mean operative time exceeded 28 min, with no evidence of plateau. Reaching a stable performance required more than twice this number of supervised cases. Whilst awaiting multicenter confirmation, our findings argue for a substantial revision of empirically set training benchmarks for THD-Anolift, with structured mentorship extended to at least 20–25 cases and routine prospective audit of personal recurrence and complication rates during the early adoption phase. We therefore propose that our results be viewed as a pragmatic reference point for training, credentialing, and audit, to be validated in larger multicenter cohorts before any prescriptive use.

It is important to note that the composite failure rate reduction from 52.2% to 25.0% did not reach conventional statistical significance (*p* = 0.080). With only 51 patients, the study was underpowered for a binary endpoint comparison, and this finding should be interpreted as a coherent trend consistent with, but not proof of, clinically meaningful improvement. The operative time reduction, by contrast, was highly significant (*p* < 0.001) and provides unambiguous evidence of a learning effect.

The overall composite failure rate of 37.3% is inflated by the inclusion of minor, self-limiting events that would correspond to Clavien-Dindo grade I (transient tenesmus, 13.7%; mild anal pain, 9.8%) and required no specific intervention. When restricted to recurrence and clinically meaningful complications, the failure rate is substantially lower (see below). When recurrence alone is considered, the 15.7% rate aligns with published systematic reviews (11–17.5%) [12, 13]. The Phase 2 recurrence rate of 10.7% narrows the gap with the 1.6% reported by Giordano and Schembari [8] in an expert series, although further improvement is expected with continued experience.

A potential confounder was the concentration of all 5 grade IV patients and a higher proportion of previous procedures in Phase 1. Beyond its statistical implications, this distribution reflects a clinical bias that we believe is important to acknowledge openly: at the start of his independent THD-Anolift experience, the operating surgeon did not preferentially select uncomplicated cases, but accepted referrals across the full spectrum of disease severity. In retrospect, current best practice for the controlled adoption of a new technique would have favoured starting with grade II–III non-recurrent cases and progressing to grade IV and revisional disease only after early-phase stabilisation. Some of the higher Phase 1 failure rate is therefore likely attributable not only to the surgeon's inexperience but also to this case-selection pattern, which our cohort cannot fully disentangle from the genuine learning effect. The RA-CUSUM addressed this by adjusting for patient-specific predicted failure probabilities, and the proficiency point remained unchanged. The logistic model found that grade IV and prior procedures had limited predictive power in this cohort (coefficients 0.10 and − 0.15), suggesting that the case-mix imbalance was unlikely to fully account for the learning curve. However, the narrow range of predicted probabilities (34–40%) also reflects the limited discriminative capacity of a model fitted on 51 observations, and this adjustment should be considered exploratory.

This study has several limitations. The retrospective, single-surgeon design limits generalizability. Fifteen percent of patients were excluded for incomplete data; this proportion introduces a potential risk of selection bias, particularly in a learning-curve study where missing data may not be randomly distributed over time. However, the chronological distribution of excluded cases (median rank 35, with only 2 of 9 occurring within the first 20 procedures), the comparable baseline characteristics between included and excluded patients, and the best-case/worst-case imputation analysis—which kept the CUSUM peak within a narrow window of cases 22–25—together suggest that loss to follow-up did not systematically displace the proficiency point. The estimated proficiency point should nevertheless be interpreted as approximate rather than absolute. Follow-up was heterogeneous, with shorter observation in Phase 2 (median 8 vs. 18 months), and late recurrences may be underestimated in recently operated patients. Patient-reported outcomes (pain scores, quality of life, satisfaction) were not systematically collected; future learning-curve studies should incorporate validated patient-reported outcome measures, as technical proficiency alone may not fully reflect the dimensions of success that matter most to patients. The composite endpoint included minor events alongside recurrence; while the sensitivity analyses on recurrence alone and on recurrence-plus-Clavien-Dindo ≥ II events both reproduced the same inflection at case 23, future studies should employ validated severity scales. The 6.7-min reduction in operative time, although highly statistically significant, is of modest absolute magnitude and should be interpreted primarily as a marker of technical fluidity rather than as a clinically transformative change in operating-room efficiency. Finally, the sample of 51 patients limits power for subgroup comparisons, and the borderline significance of the outcome comparison (*p* = 0.080) warrants cautious interpretation. Despite these limitations, this study addresses a gap explicitly acknowledged in prior publications [8, 14–16] and offers a reproducible framework for benchmarking THD proficiency.

## Conclusions

CUSUM analysis identified an inflection at approximately 23 consecutive THD-Anolift cases, supported by parallel improvement in operative efficiency and a favorable trend in clinical outcomes that was robust to sensitivity testing. Rather than defining a fixed competency threshold, these results provide an initial quantitative benchmark that may help structure supervised adoption of THD-Anolift and inform future multicenter validation studies incorporating patient-reported outcome measures and longer follow-up.

## Data Availability

The datasets generated and/or analyzed during the current study are not publicly available due to privacy and institutional restrictions but are available from the corresponding author on reasonable request.
